# Environmental Surveillance. An Additional/Alternative Approach for Virological Surveillance in Greece?

**DOI:** 10.3390/ijerph8061914

**Published:** 2011-06-01

**Authors:** Petros Kokkinos, Panos Ziros, Danai Meri, Sevasti Filippidou, Stella Kolla, Alexis Galanis, Apostolos Vantarakis

**Affiliations:** 1 Environmental Microbiology Unit, Department of Public Health, Medical School, University of Patras, Patras, GR 26504, Greece; E-Mails: pkokkin@med.upatras.gr (P.K.); zirster@gmail.com (P.Z.); danaimeri@yahoo.gr (D.M.); sphilippidou@upatras.gr (S.F.); 2 Department of Molecular Biology and Genetics, Democritus University of Thrace, Alexandroupolis, GR 68100, Greece; E-Mails: stkolla@hotmail.com (S.K.); agalanis@mbg.duth.gr (A.G.)

**Keywords:** sewage, adenovirus, hepatitis A virus, hepatitis E virus, norovirus, polyomavirus, environmental surveillance

## Abstract

The detection of viruses in the sewage of an urban city by nucleic acid amplification techniques allows the identification of the viral strains that are circulating in the community. The aim of the study was the application of such detection which gives useful data on the distribution, spread, and frequency of these viruses, supporting epidemiological studies of the related viral infections. A two year (2007–2009) survey was conducted in order to evaluate the presence of human adenoviruses (hAdV), hepatitis A viruses (HAV), hepatitis E viruses (HEV), noroviruses (NoV), and human polyomaviruses (hPyV) in sewage samples collected from the inlet of a municipal biological wastewater treatment plant located in southwestern Greece. PCR methods were used for this survey. In total, viruses have been detected in 87.5% (42/48) of the analyzed sewage samples. Analytically, DNA viruses, hAdVs and hPyVs have been detected in 45.8% (22/48) and 68.8% (33/48) of the samples, respectively. As it concerns RNA viruses, HAV was detected in 8.3% (4/48), NoVs in 6.3% (3/48), while HEV has not been detected at all. After sequencing, AdVs were typed as Ad8, Ad40 and Ad41, while both JC and BK hPyVs have been recognized. All NoVs have been identified as GII4, while HAV was typed as genotype IA. Similar long-term studies could be undertaken in countries such as Greece in order to offer a valuable and complementary tool to current problematic epidemiological surveillance systems. This study demonstrates the advantages of environmental surveillance as a tool to determine the epidemiology of viruses circulating in a given community. To our knowledge this was the first of its kind study performed in Greece in order to establish this new way of surveillance.

## 1. Introduction

The bibliography has been enriched the last few years by several studies which have demonstrated the advantage of environmental surveillance as an additional tool to determine the epidemiology of different viruses circulating in a given community [1–8]. Environmental poliovirus surveillance (EPS) systems have been introduced as powerful tools for surveillance of poliovirus circulation and re-emergence of virulent poliovirus from attenuated vaccines in the absence of paralytic poliomyelitis, especially in populations with high vaccine coverage [9]. Recently, environmental poliovirus surveillance programs have been introduced to South Africa, Estonia, Slovakia, Japan, Russia, and Finland [1,5,9]. The availability of improved detection techniques, combined with an increased awareness of gastroenteritis-causing viral pathogens, has also led to the establishment of surveillance systems in various countries, since other enteric viruses responsible for gastroenteritis and hepatitis have replaced enteroviruses as the main target for detection [8,10].

The enteric viruses found in human stools belong to more than 140 types of which adenovirus (AdV), hepatitis A virus (HAV), norovirus (NoV) genotype I and II, rotavirus (RV) and enterovirus (EV) are those most often detected in the environment [11–15]. HAdVs are associated with sporadic cases and occasional outbreaks of gastroenteritis. Out of the six subgroups, AdVs of subgroup F (enteric serotypes 40 and 41) are estimated to be associated with 5–20% of acute gastroenteritis cases among infants and young children [16]. Hepatitis A represents worldwide around 50% of the total hepatitis cases and hepatitis A virus has been linked to several waterborne outbreaks. Hepatitis E is less frequent than hepatitis A, and in industrialized countries is thought to be spread zoonotically, principally from swine [8]. NoVs are an important cause of epidemic acute gastroenteritis, and waterborne outbreaks of NoV-associated gastroenteritis are well documented [7]. The human polyomavirus has been shown to be present in high concentrations in the sewage, and its specificity as a human virus may be useful as a marker for fecal pollution of anthropogenic origin [17].

Environmental surveillance can provide valuable supplementary information, particularly in urban populations with absent or questionable surveillance, when persistent virus circulation is suspected, or frequent virus re-introduction is perceived [18]. In Greece, currently, there is no environmental surveillance system in action, while the national surveillance system for gastroenteric viruses is inadequate or absent.

In the present study, a two-year environmental survey (2007–2009) was conducted in order to evaluate the presence of human adenoviruses (hAdV), hepatitis A viruses (HAV), hepatitis E viruses (HEV), noroviruses (NoV), BK (BKPyV) and JC (JCPyV) human polyomaviruses (hPyV) in sewage samples collected from the inlet of a municipal biological wastewater treatment plant located in southwestern Greece. The study aimed to enrich the poor data on environmental virological studies in Greece, demonstrate the benefit of environmental surveillance as a tool to determine the epidemiology of viruses circulating in a given community, and to underline the need for the design and support of similar long-term studies in our country, in order to support the absent national surveillance system for gastroenteric viruses.

## 2. Experimental Section

### 2.1. Wastewater Treatment Plant and Sampling

The municipal wastewater treatment plant of the present study receives urban sewage from the city of Patras. The municipality has 171,616 inhabitants (census of 2001) and it is located in southwestern Greece. The plant is officially registered as a secondary treatment plant with anaerobic digestion of the sludge. It treats 38,000 m^3^ of urban sewage per day. The wastewater effluents are discharged into the Patraikos Gulf. From November 2007 to July 2009, 48 samples of untreated sewage were collected from the municipal sewage treatment plant. The samples were transferred to the laboratory into a coolbox and they were immediately subjected to virological analysis for the detection of human AdVs, NoVs, HEV, HAV, BKPyV and JCPyV.

### 2.2. Sample Concentration, Viral Extraction and Biomolecular Analysis

Sewage samples were concentrated to a final volume of 1 mL PBS after centrifugation at 220,000 g for 1 h, according to previously published protocols [16,19,20]. Viral nucleic acids were extracted from concentrated samples using the QIAamp Viral RNA mini-kit according to the manufacturer’s instructions, (QIAcube, Qiagen, USA). Reverse transcription polymerase chain reaction (RT-PCR) and nested PCR techniques have been used for the detection of human AdVs, HAV, HEV, NoVs, JC and BK hPyVs, according to previously published protocols [19–21].

### 2.3. Sequence Analysis

Positive PCR products were purified using the QIAquick PCR purification kit (Qiagen, USA) and confirmed by sequencing (Sequencing unit, School of Medicine, University of Thessaly, Greece). The obtained nucleotide sequences were analyzed by BLAST program at the NIH web-site (NCBI, National Centre for Technology Control, NIH, USA), and were compared with each other and with other published sequences. Multiple alignments were performed with the Clustal X program.

## 3. Results

**Figure 1.**
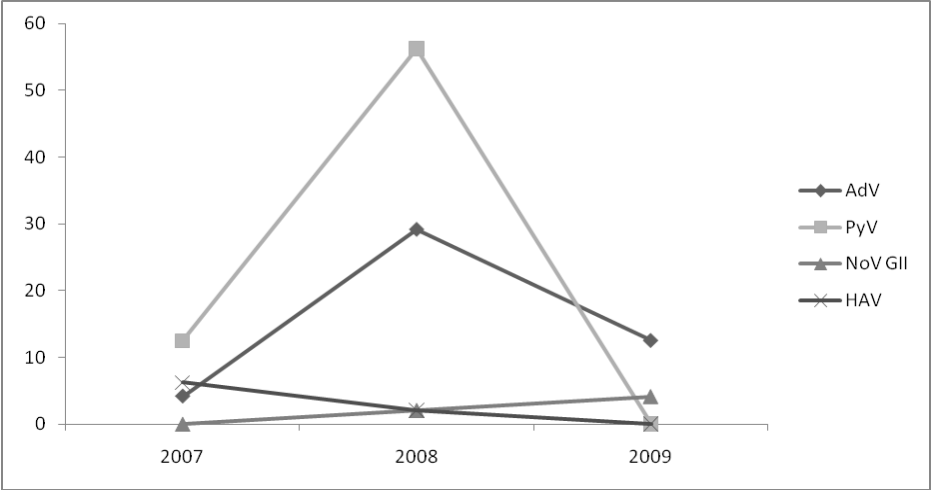
Annual percentage (%) detection of viruses in forty-eight (48) analyzed sewage samples from the inlet of the wastewater treatment plant. AdV: adenoviruses, PyV: polyomaviruses, NoV GII: noroviruses, HAV: hepatitis A virus.

**Figure 2.**
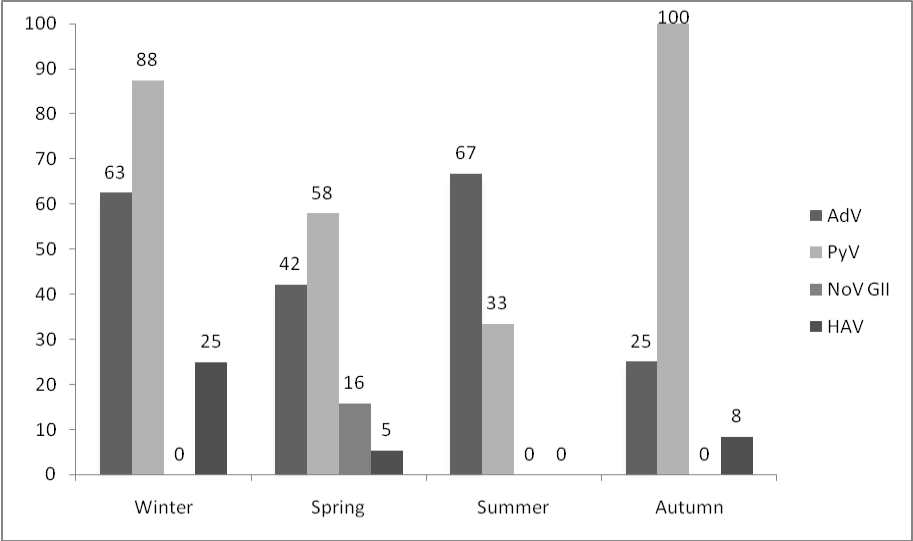
Seasonal percentage (%) detection of viruses in the forty-eight (48) analyzed sewage samples. AdV: adenoviruses, PyV: polyomaviruses, NoV GII: noroviruses, HAV: hepatitis A virus.

A two year (2007–2009) survey was conducted to examine the HAV, hAdVs, HEV, NoVs, BKPyVs and JCPyVs presence in sewage samples collected from a biological wastewater treatment plant, located at the city of Patras. The yearly sampling is shown in Figure 1. In total, viruses have been detected in 87.5% (42/48) of the sewage samples collected from the plant’s inlet. Analytically, DNA viruses, adenoviruses, have been detected in 45.8% (22/48) of the samples and PyVs have been detected in 68.8% (33/48) of the sewage samples. As it concerns RNA viruses, hepatitis A viruses were detected in 8.3% (4/48), noroviruses in 6.3% (3/48), while HEV has not been detected at all. After sequencing, AdVs were typed as Ad8, Ad40 and Ad41, while both JC and BK hPyVs have been recognized. All NoVs have been identified as GII4, while HAV was typed as genotype IA. Also the seasonal presence of the various types of viruses is described in Figure 2.

## 4. Discussion

During the last years, more attention has been focused on the sewage virological quality, the risk of virus-associated waterborne illness, the need for routine monitoring viral contamination and the environmental surveillance through the analysis of sewage [12,22,23]. To enrich the poor existing virological data and underline the need of environmental surveillance programs in Greece, a two years (2007–2009) survey was conducted to examine the HAV, hAdVs, HEV, NoVs, and hPyVs presence in sewage samples collected from a biological wastewater treatment plant, located at the city of Patras.

Recently, in an attempt to characterize the major enteric virus (enterovirus, rotavirus, norovirus, astrovirus and adenovirus) presented in the sewage of Greater Cairo, the co-circulation of enteric viruses in sewage and in the population has been reported [24]. A prospective study to characterize the main human enteric viruses able to persist in sewage samples and to establish the correlation between environmental strains and viral infantile diarrhoea observed in the Monastir region (Tunisia), was performed by Sdiri-Loulizi and colleagues. Group A rotavirus, norovirus and enteric adenovirus were detected in 80 (32%), 11 (4.4%) and 1 (0.4%) sewage samples, respectively, addressing the potential health risks associated with transmission of human enteric viruses through water-related environmental routes [25]. Interestingly, the analysis of our norovirus strains revealed a high degree of homology with the norovirus strains of this study. GII.4 is the predominant genotype worldwide [26].

Limited data on enteropathogenic viruses are available in Greece, because there is no formal surveillance system for viral gastroenteritis. The role of enteric viruses as a cause of gastroenteritis in northwestern Greece was investigated by a 6-year study of stool samples from children hospitalized for acute gastroenteritis. Rotaviruses, noroviruses, adenoviruses and astroviruses were detected in 21.35%, 4%, 3.5% and 2.35%, respectively. Noroviruses were confirmed as the second most common viral agent after Rotavirus among hospitalized children with acute gastroenteritis. Astrovirus and adenovirus infection rates were also comparable. Although enteric adenovirus types 40 and 41 predominated, non-enteric subgenera (A and C) were also causally implicated [27]. In our study, the analysis of sewage samples mainly revealed adenoviruses types 40 and 41, and Ad8 in few cases as well.

Molecular methods for the detection and typing of hepatitis A virus (HAV) strains in sewage were applied to determine its distribution in Cairo and Barcelona [8]. That study revealed the occurrence of different patterns of hepatitis A endemicity in each city, while the circulating strains were characterized as being of genotype IB. In the past, Greece was characterized as a region with an intermediate prevalence rate for hepatitis A. Recent seroepidemiologic data regarding this disease are severely limited. According to the latest national cross-sectional seroprevalence survey of hepatitis A among Greek children, hepatitis A is intermediate endemic in Greece and in light of that study the National Advisory Committee for Immunization has included the hepatitis A vaccine in the GNIP from January 2008 [28]. In our study, HAV was detected in four (out of forty-eight) sewage samples. In a previous study, one of five sewage samples from Patras tested positive for HAV [29].

Hepatitis E virus is the causative agent for enteric non-A, non-B hepatitis. HEV is considered an emerging pathogen in industrialized countries. A molecular HEV screening performed on raw sewage samples from different wastewater treatment plants yielded positives at 16%, evenly distributed throughout Italy [30]. In a study aimed to determine the prevalence of anti-HEV among haemodialysis patients of a semi-rural region in central Greece showed that the prevalence of anti-HEV was greater than in healthy blood donors [31]. In the last few years, some HEV strains associated with sporadic acute hepatitis have been isolated from human serum samples in North America and Europe (*i.e.*, Italy, Greece, Spain, and the United Kingdom). The level of infection in regions where HEV is considered non endemic by analyzing the excreted virus in the urban sewage of diverse geographic areas has been previously reported [29]. Interestingly, in that study, HEV RNA was not detected in any of the sewage samples derived from Patras [29]. In our study, none of the sewage samples was found positive for HEV.

The first description of the distribution of human polyomaviruses in urban sewage performed by Bofill-Mas, showed that these viruses are spread in high concentrations in the sewage of different geographical areas, are present in contaminated environments, and may be useful as a marker for fecal pollution of anthropogenic origin [17]. Polyomavirus BK infection in Greek renal transplant recipients has been reported by Zavos [32]. There is no previous data on the presence of JC or BK PyVs in sewage in Greece. In our study, JC and BK PyVs have been detected in 68.8% of the samples.

A sewage surveillance system has been shown to be more sensitive than reporting of clinical cases of serious illness in a community. It was also demonstrated that pathogens can be greatly retarded or protected in a sewage system allowing a detection time over many days for a one-time release into a sewage system. Finally, it was shown that infectivity assays have the ability to detect one infected person in 10,000 individuals. Sewage surveillance may detect the presence or increased amount of infections from enteric pathogens excreted in the feces or urine during infection [33]. The sensitivity of a sewage surveillance system will depend on several important factors, including the amount and duration of the agent released into the sewers, the frequency of monitoring, and the sensitivity of the monitoring method. This monitoring is especially useful when combined with other components of the qualitative microbial risk assessment (QMRA) framework such as modeling of sewage dispersion, back calculation of contaminant point of introduction, and calculations of the health risk [33].

Data from the occurrence of viruses in raw sewage may provide an overview of the epidemiology of virus infections circulating in the community, and at the same time, reveal the occurrence of asymptomatic infections [8,23]. Our study demonstrates the benefit of environmental surveillance as a tool to elucidate the molecular epidemiology and circulation of viruses. Since there is actually no environmental surveillance program in action in Greece and the epidemiological surveillance system is problematic, we propose that similar long-term studies should be undertaken in countries such as ours to offer a valuable and complementary tool to epidemiological surveillance systems.
